# Regular Nonsteroidal Anti-Inflammatory Drug Use Increases Stress Fracture Risk in the General Population: A Retrospective Case-Control Study

**DOI:** 10.1155/2024/7933520

**Published:** 2024-10-12

**Authors:** Alexandra Ciuciu, Christopher Mulholland, Michael A. Bozzi, Chris C. Frymoyer, Leonardo Cavinatto, David Yaron, Marc I. Harwood, Jeremy D. Close, Christopher J. Mehallo, Ryan E. Tomlinson

**Affiliations:** ^1^Department of Orthopaedic Surgery, Thomas Jefferson University, Philadelphia 19107, PA, USA; ^2^Department of Orthopaedic Surgery, William Beaumont University Hospital, Corewell Health, Royal Oak 48073, MI, USA; ^3^Department of Sports Medicine, The Rothman Orthopaedic Institute, Philadelphia 19107, PA, USA

## Abstract

Previous studies have shown that the use of nonsteroidal anti-inflammatory drugs (NSAIDs) is associated with increased stress fracture risk. This phenomenon has been studied predominantly in high-activity individuals, so data regarding the general population are limited despite the substantial economic and resource burden of stress fracture injuries within the general US population. Furthermore, our preclinical studies demonstrate that regular use of NSAIDs also diminishes the intrinsic ability of bone to resist fracture. To determine the association of regular NSAID use with stress fractures in the general population, we surveyed subjects presenting with either stress fracture or uncomplicated ankle sprain to assess their use of NSAIDs over the three months before their injury. We hypothesized that subjects with stress fractures would have increased regular NSAID usage as compared to controls. Subjects diagnosed with a stress fracture (*n* = 56) and subjects with uncomplicated ankle sprains (*n* = 51; control) were surveyed about their NSAID use at the time of their diagnosis and in the previous three months using a questionnaire based on the National Health and Nutrition Examination Survey (NHANES). Subjects were surveyed in person on the day of their injury diagnosis or by phone within 30 days of their diagnosis. Fisher's exact test was used to determine significant differences in NSAID usage between stress fracture and control subjects. Subjects diagnosed with stress fractures had a statistically significant increase in both current use (*p*=0.03) and regular use (*p*=0.04) of ibuprofen/naproxen/celecoxib as compared to control subjects. There were no significant differences in the use of aspirin, acetaminophen, or prescription medications containing acetaminophen between groups. Consistent with previous clinical reports, we observed a strong correlation between regular ibuprofen/naproxen/celecoxib use and stress fracture incidence in the general population. These results indicate that patients at high risk of stress fracture should avoid regular use of ibuprofen, naproxen, or celecoxib.

## 1. Introduction

Nonsteroidal anti-inflammatory drugs (NSAIDs) are common medications used to relieve pain, fever, and inflammation. Globally, NSAIDs are used by more than 30 million people daily and are often overused [1]. There is a growing body of preclinical evidence indicating that regular NSAID use diminishes bone formation in response to mechanical forces in areas of high strain, reduces bone toughness (the work required to fracture a bone), and impairs fracture healing [2–7]. Despite these observed negative effects of NSAIDs in bone, our understanding of the mechanisms behind the effects and which drugs cause them is incomplete. NSAIDs function by inhibiting the cyclooxygenase (COX) enzymes COX1 and/or COX2 from producing prostaglandins that can trigger pain or inflammation, but certain prostaglandins, particularly prostaglandin E2 (PGE2), are involved in strain-adaptive bone remodeling and fracture healing [5, 6, 8].

Strain-adaptive bone remodeling and microdamage repair are bone functions that work in tandem to prevent stress fractures, which are painful fatigue injuries commonly caused by repetitive, submaximal forces on bone that from an osseous stress response that can propagate into larger fractures [9, 10]. Stress fractures are commonly diagnosed in active individuals, such as athletes, dancers, and military personnel, and these injuries represent up to 20% of all injuries diagnosed in sports medicine clinics [2, 9–11]. The treatment for a stress fracture often involves protected weight bearing and immobilization and can even require surgery. This imposes a large burden on the individual and the healthcare system. As a result, determining the factors that increase stress fracture incidence is a critical research goal.

Two recent studies have provided some mechanistic insights regarding the relationship of NSAIDs and stress fractures. First, one study assessed NSAID usage in US Army Soldiers [2]. Here, the investigators observed a 2.9-fold increase in stress fracture incidence associated with NSAID prescription in the general army population and a 5-fold increase in incidence for those in basic combat training. These results imply a general risk of NSAIDs in this active population that is increased during scenarios of particularly strenuous physical activity. Next, our laboratory investigated the impact of these NSAIDs in a preclinical mouse model [6]. Here, we observed a significant impact of the NSAID naproxen to decrease load-induced bone formation, as observed with NSAID administration in previous studies, as well as a novel deleterious effect on bone toughness [3, 5, 8, 12, 13]. We expect that both effects would increase stress fracture incidence, particularly when taken regularly.

Unfortunately, most studies on stress fracture incidence have been limited to athletes or military personnel, rather than the general population [9, 10]. Therefore, the objective of this study was to quantify the association between regular NSAID use and stress fracture incidence in the general population. To do so, we used a retrospective case-control study of subjects diagnosed with a stress fracture as compared to subjects diagnosed with an uncomplicated ankle sprain (control). Our overall hypothesis was that subjects presenting with a stress fracture would have significantly higher regular NSAID use as compared to control subjects.

## 2. Materials and Methods

### 2.1. Recruitment

This study protocol was approved by the IRB of Thomas Jefferson University (IRB #17D.318). Male and female participants (age range: 18–83 years) recruited for this study between February and May of 2018 were diagnosed in our clinic with a stress fracture that was confirmed using MRI without contrast (ICD10 codes M84.30-M84.38). A total of 125 subjects diagnosed with stress fractures were considered, of which 69 subjects declined to participate. Subjects diagnosed with uncomplicated ankle sprains (ICD10 code S93.4) were used as controls and recruited between October of 2020 and February of 2021. Subjects with uncomplicated ankle sprains were chosen as controls because they were diagnosed with a musculoskeletal injury and were likely to be taking NSAIDs for musculoskeletal pain relief but did not have a bone injury. Though ankle sprains and stress fractures are different injuries with distinct pathology and treatment, the comparison between a bone injury and a soft-tissue injury allows for the identification of any correlation that exists between NSAID use and bone injuries specifically. A total of 214 ankle sprain subjects were considered, of which 163 declined to participate and 1 was excluded due to a preexisting condition causing chronic pain and frequent NSAID use. Overall, 56 subjects with stress fractures and 51 subjects with uncomplicated ankle sprains were recruited to participate in the study. A detailed description of subjects that were included, declined to participate, and excluded due to preexisting conditions is available in a CONSORT flow diagram (Supplementary Figure 1).

### 2.2. Questionnaire

To participate in this study, subjects were asked to complete a questionnaire in person or over the phone within 30 days of their injury diagnosis. All responses were recorded post-diagnosis of either a stress fracture or uncomplicated ankle sprain. First, each subject provided background information about their gender, age, height, weight, zip code, race, and the location of their stress fracture (if applicable). Next, their current and regular use of NSAIDs was determined using a series of questions taken from the National Health and Nutrition Examination Survey (NHANES) [14]. Specifically, we collected the following variables: ASPIRIN, ASPIRIN3M, ADVIL, ADVIL3M, ACETOCT, ACETOCT3M, ACETPR, and ACETPR3M (Table 1). Subjects were asked if they took any of the drugs within the defined categories for at least three days within the last week, which was defined as “current use.” If subjects answered yes, they were then asked if they have taken that drug regularly (defined as at least 3 times per week) for the past three months, which was defined as “regular use.”

### 2.3. Statistical Analysis

Before analysis, data were de-identified and the results were analyzed for current and regular drug use between stress fracture and control subjects. Statistical analysis was performed using Fisher's exact test, unpaired Student's *t*-test, and Chi-square test in GraphPad Version 8.0.0. Statistical tests evaluating the number of males and females in the total study and the number of subjects in each drug use category were done using Fisher's exact test. Fisher's exact test was used to compare these categories to provide the same statistical comparison as several groups contained too few subjects to use other contingency tests. Fisher's exact test assumes independence of observations, unpaired data, and that the data contain number of subjects. The study design of choosing participants from the same clinic and collecting information using a standard questionnaire attempts to control for independence of observation, and all contingency comparisons were done on unpaired data using number of subjects in each category. A significant difference was considered as any Fisher's exact test that resulted in a *p* value less than 0.05. Statistical tests regarding mean age and BMI were done using unpaired Student's *t*-test with significance defined as any *p* values less than 0.05. Finally, race demographics between stress fracture and uncomplicated ankle sprain groups were compared using a Chi-square test for trend with a significance threshold of a *p* value below 0.05.

## 3. Results

### 3.1. Subject Demographics

Of the 56 stress fracture subjects and 51 uncomplicated ankle sprain (control) subjects screened for enrollment, one control subject was excluded due to chronic pain that resulted in regular NSAID use. With regard to cohort characteristics (Table 2), there were no significant differences between groups in mean age, sex, or race, with a small but significant difference in body mass index (BMI). Although not significantly different between groups, subjects in both cases and controls were predominantly female and white. The location of stress fractures in this cohort (Table 3) was primarily in the pelvis/femur (32%), the tibia/fibula (32%), and the ankle/foot/toe (30%).

### 3.2. Current NSAID Use

**Figure 1:**
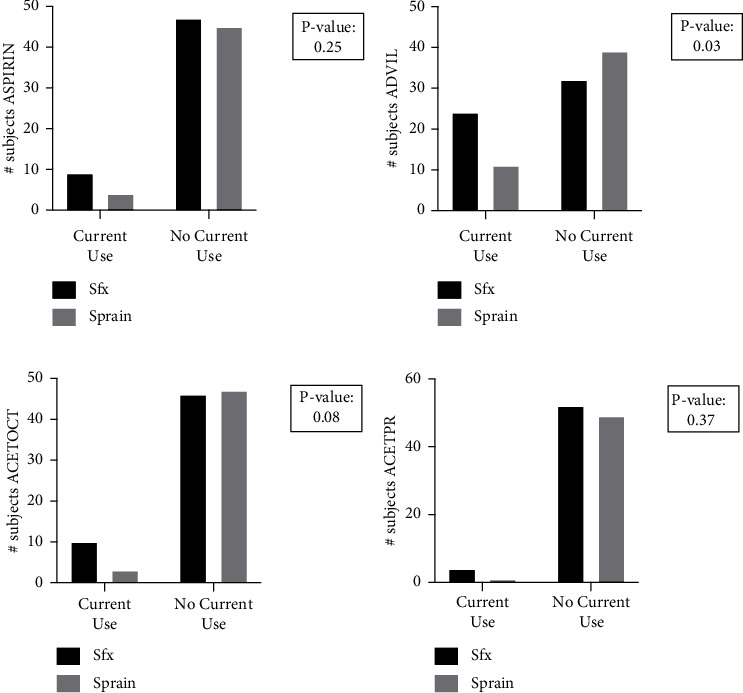
Current drug use by category. Subjects diagnosed with stress fractures or uncomplicated ankle sprains answered yes or no to survey questions regarding their current analgesic drug use, defined as 3 or more times per week. Results were divided into (a) ASPIRIN, (b) ADVIL, (c) ACETOCT, and (d) ACETPR. There was a significant (*p* < 0.05) increase in the amount of stress fracture subjects currently using drugs within the ADVIL category when compared to control subjects.

**Figure 2:**
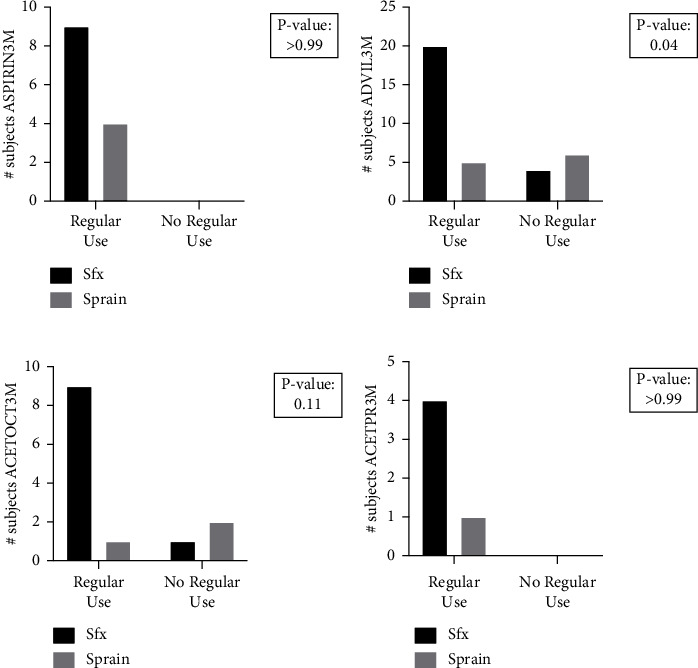
Regular drug use by category. Subjects diagnosed with stress fractures or uncomplicated ankle sprains answered yes or no to survey questions regarding their regular analgesic drug use, defined as 3 or more times per week for the past 3 months. Results were divided into (a) ASPIRIN3M, (b) ADVIL3M, (c) ACETOCT3M, and (d) ACETPR3M. There was a significant (*p* < 0.05) increase in the amount of stress fracture subjects currently using NSAIDs within the ADVIL3M category when compared to control subjects.

First, we examined NSAID use at time of presentation with injury (Table 4). Here we observed that subjects with stress fractures had a significantly higher incidence of current use of drugs within the ADVIL category (*p*=0.025) as compared to control subjects (OR: 2.7, 95% CI: 1.1–6.1) (Figure 1). There were no significant differences between the percentage of subjects in the ASPIRIN, ACETOCT, or ACETPR groups. Nonetheless, we observed that subjects with stress fractures had significantly higher current use of any queried drug (*p* < 0.0001) as compared to control subjects (OR: 8.5, 95% CI: 3.5–19) (Figure 2).

### 3.3. Regular NSAID Use

**Figure 3:**
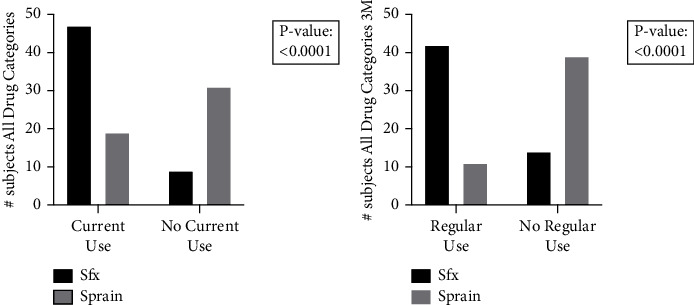
Current and regular use of all surveyed drugs. (a) Total subject responses were pooled to quantify the number of stress fracture subjects compared to controls currently using any of the surveyed drugs. A significantly (*p* < 0.05) higher amount of stress fracture subjects currently used any of the surveyed drugs when compared to control subjects. (b) Total subject responses were pooled to quantify the number of stress fracture subjects compared to controls using any of the surveyed drugs regularly, defined as three or more times per week for three months. A significantly higher amount of stress fracture subjects regularly used any of the surveyed drugs when compared to control subjects. Overall, the use of any NSAID or acetaminophen-containing drug included in this surveyed was correlated to stress fracture diagnosis.

Next, we considered NSAID use in the previous three months before presentation with injury (Table 5), in which regular NSAID use was defined as three or more times per week during the three-month period. Here again, stress fracture subjects had a significantly higher incidence of regular use of drugs within the ADVIL category (*p*=0.041) as compared to the control subjects (OR: 6.0, 95% CI: 1.2–26) (Figure 3). There were no significant differences between subjects in stress fracture and control groups for ASPIRIN3M, ACETOCT3M, or ACETPR3M. Finally, we analyzed pooled responses for regular use of any NSAID. We observed that stress fracture subjects had a significantly higher incidence of regular use of any queried drug (*p* < 0.0001) as compared to control subjects (OR: 10.6, 95% CI: 4.1–25) (Figure 2).

## 4. Discussion

In this study, we examined the relationship between regular NSAID use and stress fracture incidence. To do so, we assayed subjects from a single clinic with stress fracture or uncomplicated ankle sprain using the NHANES questionnaire. We found that regular use of ibuprofen, naproxen, and/or celecoxib was highly associated with stress fracture diagnosis. In contrast, we found no significant correlations between stress fracture diagnosis and the use of drugs containing acetaminophen, aspirin, or prescription drugs containing acetaminophen. Nonetheless, the pooled analysis of all drugs surveyed did indicate a strong association between stress fracture diagnosis and NSAID or acetaminophen-containing drug usage. In total, these results are consistent with our overall hypothesis and suggest that the surveyed drugs should not be used regularly by individuals at higher risk of stress fracture.

This study contributes to a growing body of evidence that NSAIDs are associated with increased stress fracture risk [5]. Importantly, these results from the general population are consistent with previous findings from a larger retrospective study of US Army Soldiers, in which naproxen and ibuprofen were associated with the highest stress fracture incidence [2]. In addition to being the most popular NSAID category in our cohort, the drugs within the ADVIL category are potent COX2 inhibitors. COX2 is responsible for the synthesis of prostaglandin E2 (PGE2), which has been shown in preclinical and clinical models to contribute to strain-adaptive bone remodeling and fracture healing [3, 5, 6, 8]. Thus, potent COX2 inhibitors, particularly when taken regularly, could diminish the amount of new bone formed in response to mechanical loading because of impaired PGE2 synthesis. Furthermore, decreased strain adaptation has been shown to decrease bone resistance to fatigue injuries like stress fractures [15, 16]. This mechanism is thought to be the causative factor driving the association between regular NSAID use and stress fracture incidence in highly active individuals [2, 5]. Interestingly, previous studies have shown that different levels of PGE2 are produced when someone is at rest as compared to when they are active, suggesting that PGE2 levels of a highly active individual may be different than someone in the general population [17]. These differences in PGE2 and other prostaglandins could indicate that NSAIDs influence bone in the general population differently than in other population groups. Although our results are consistent with previous studies, we cannot conclude that any one of the drugs in the ADVIL variable (ibuprofen, naproxen, and celecoxib) is necessarily the most deleterious NSAID for bone health. In fact, our study reports that the odds for a subject with a stress fracture to have been taking any NSAID regularly (OR: 10.6, 95% CI: 4.1–25) are not significantly different than the odds for a subject with stress fracture to have been taking just the ADVIL NSAIDs regularly (OR: 6.0, 95% CI: 1.2–26). Since ibuprofen, naproxen, and celecoxib have substantially different COX selectivity, typical dosages, and strength of COX inhibition, these NSAIDs should be evaluated separately in future experimental work.

Our study did not report any significant correlation between current or regular use of aspirin, acetaminophen, or prescription medications containing acetaminophen and stress fracture incidence. This lack of correlation could be due to the action of these NSAIDs. Aspirin causes irreversible inhibition of the COX enzymes and the formation of several metabolic byproducts that are produced quickly after ingestion, which may limit its effects in bone compared to other NSAIDs [18]. Contrastingly, acetaminophen is not an NSAID, but its analgesic and antipyretic actions in humans are believed to be mediated through binding with the COX enzymes. Also, acetaminophen may inhibit strain-adaptive bone remodeling and fracture healing similarly to NSAIDs [18, 19]. Finally, the prescription medications containing acetaminophen are less accessible than over-the-counter analgesic medications and had the least number of users in both injury categories. Despite these contributing factors, the effects of these medications should continue to be investigated to minimize stress fracture risk in the general population. In particular, previous studies have demonstrated effects of NSAIDs and acetaminophen that are independent of COX inhibition, so directly assessing the effects of these medications on bone overuse injuries, rather than COX-dependent signaling, is required [20, 21].

Both animal and human studies indicate a correlation between regular NSAID use and stress fracture incidence, but NSAIDs continue to be an effective and accessible means of pain control in clinical settings [5]. In fact, some clinical early discharge and enhanced recovery protocols rely on NSAID use to manage patient pain without the use of addictive opioids [22, 23]. These protocols not only effectively manage patient pain but also can reduce hospital lengths of stay, minimizing the risk of further complications [24]. Thus, future, highly-powered studies are needed to describe the mechanisms by which NSAIDs may impact bone to inform NSAID use in the general population.

This study has several limitations. The first limitation is the relatively small subject cohort that was predominantly female, white, and older—the population most at risk for osteopenia and osteoporosis. Since the effects of NSAIDs may be sex, race, and age dependent, this may limit the application of our findings to a larger general population [25]. Given that sex, race, and age were not statistically significantly different across stress fracture and uncomplicated ankle sprain groups, the results of this study are likely applicable for individuals of the general population who are female, white, and about 50 years old. The subjects participating in this study were not actively recruited for a stress fracture study but retrospectively assayed about drug use after coming in voluntarily to the clinic for injury diagnosis. Since females have multiple life events that can influence the risk of musculoskeletal injury (e.g. puberty, pregnancy, and menopause), it is not surprising to find that most subjects recruited from clinic visits for musculoskeletal injuries were female [26]. The location and timing of subject recruitment can also influence the sex, race, and ages of subjects, and thus any smaller scale, retrospective study is likely to survey only a small demographic. This limited application highlights the need for future, large-scale studies done in the general population to determine whether there exists a correlation between NSAID use and stress fracture injury across sexes, races, and ages. Moreover, the physical activity level of the subjects was not assayed. Stress fractures form from repetitive strains on the bone over an extended period, so subjects may have an injury for some time before going to the clinic. In contrast, an uncomplicated ankle sprain is symptomatic immediately. This difference in injury pathology between the study cohorts can mean that activity level is a confounding variable to the pain experienced by each subject, which could influence NSAID use patterns. Future studies should include a measurement of activity levels and compare cases with similar levels of exercise to ensure that lifestyle differences do not exacerbate subject injuries and encourage more NSAID use. In fact, the only significant difference between stress fracture and control subjects in this study was BMI, with the ankle sprain cohort having a slightly higher average BMI (30.5 kg/m^2^) than the stress fracture cohort (27.1 kg/m^2^)—perhaps indicating an overall higher activity level in the stress fracture subjects [27]. However, BMI is not always an accurate indication of activity levels and assaying exercise more directly would benefit future studies of the general population. In addition to the central role of physical activity on stress fracture incidence, previous studies have shown that administration of NSAIDs before or after exercise affects the skeletal consequences [3, 5, 12, 13]. Thus, monitoring when NSAIDs are taken relative to exercise may also be an important consideration. A limitation of the retrospective, case-control study design is determining causality between NSAID use and stress fracture incidence. Specifically, it is not known whether subjects in the stress fracture cohort developed their injury before or after initiating regular NSAID use. To limit potential recall bias, subjects were asked about their NSAID usage as soon after their diagnosis as possible. Furthermore, the questionnaire focused on time periods of NSAID use, either the last week or a prolonged three-month period, which are more likely to be remembered than specific dates. An additional limitation is the lack of information regarding subject diet, supplement use, and other medication use. These factors can have profound effects on bone health, so future studies should consider capturing these data [28]. A final limitation is that a stress fracture is an overuse injury that often takes longer to form and diagnose than an ankle sprain, which is often due to a single traumatic event. This fact coupled with the regular access to over-the-counter drugs including ibuprofen, naproxen, and acetaminophen means that it is possible that subjects were self-medicating with the surveyed drugs before their official diagnosis. Additionally, subjects have more access to over-the-counter medications than prescribed drugs, which may have led to the diminished sample sizes within the ACETPR group. Future studies should consider drug accessibility as a factor when recruiting subjects. Despite these limitations, the overall study conclusions are consistent with previous clinical and preclinical studies regarding the strong correlation between NSAIDs and stress fracture risk.

## 5. Conclusion

Our study found a significant correlation between the regular use of ibuprofen, naproxen, and/or celecoxib with stress fracture diagnosis in the general population. This finding is consistent with preclinical studies in animal models as well as clinical data from US Army Soldiers [2, 5, 6, 11]. NSAID use likely increases the risk of stress fracture by decreasing inherent toughness and diminishing the ability of bone to repair microdamage and remodel in response to mechanical loading. Future studies should include observation of a more diverse study population as well as mechanistic experiments to investigate the effect of individual NSAIDs. Nonetheless, this study is one of a growing body of literature that has linked stress fracture incidence with NSAID usage. As a result, it is recommended that patients at high risk for stress fracture refrain from regular NSAID usage.

## Figures and Tables

**Table 1:** Medication variables.

Drug category	Included drugs
ASPIRIN, ASPIRIN3M	Aspirin, Bayer, Bufferin, Excedrin
ADVIL, ADVIL3M	Advil, Ibuprofen, Motrin, Nuprin, Aleve, Naprosyn, Naproxen, Celebrex, Celecoxib
ACETOCT, ACETOCT3M	Tylenol, Tylenol PM, Nyquil, Theraflu, Excedrin, Alka Seltzer Plus, Midol, Acetaminophen
ACETPR, ACETPR3M	Vicodin, Percocet, Endocet, Tylenol with Codeine, Fioricet

These variables were previously defined in the NHANES questionnaire.

**Table 2:** Subject demographics.

Characteristics		Stress fracture, *n* = 56	Ankle sprain (control), *n* = 50	*p* value	Significance
Female sex—%		73%	64%	0.40	ns

Mean age (SD)—years		51.4 (19.5)	51.2 (13.9)	0.96	ns

Mean BMI (SD)—kg/m^2^		27.1 (5.5)	30.5 (8.7)	0.02	^∗^

Race	White	51	41	0.53	ns
	Black	2	3		
	Asian	0	2		
	Hispanic	0	2		
	Other	0	1		
	Prefer not to answer	3	1		

This table includes the demographic distribution of subjects within each injury category. Fisher's exact test (sex), unpaired Student's t-test (age and BMI), and Chi-square test for trend (race) were used to determine *p* values. ^∗^*p* < 0.05; ns = not significant.

**Table 3:** Stress fracture location.

Location of stress fracture	Stress fracture, *n*	ICD-10 code
Shoulder	1	M84.31
Humerus	1	M84.32
Ulna or radius	0	M84.33
Hand or finger	0	M84.34
Pelvis or femur	18	M84.35
Tibia or fibula	18	M84.36
Ankle, foot, or toe	17	M84.37
Other	1	M84.38

This table includes the reported locations of stress fractures of subjects in the stress fracture injury group.

**Table 4:** Current drug use.

Drug category	Stress fracture, *n*			Ankle sprain (control), *n*			*p* value	Significance
	Yes	No	Not sure	Yes	No	Not sure		
ASPIRIN	9	47	0	4	45	1	0.25	ns
ADVIL	24	32	0	11	39	0	0.03	^∗^
ACETOCT	10	46	0	3	47	0	0.08	ns
ACETPR	4	52	0	1	49	0	0.37	ns
All drug categories	47	9	0	19	30	1	<0.0001	^∗∗∗∗^

This table includes the enumerated responses of subjects within each injury group regarding current drug use defined as three or more times per week at the time of inquiry. ^∗^*p* < 0.05; ns = not significant. ^∗∗∗∗^ is defined as significant with a *p* value <0.0001.

**Table 5:** Regular drug use.

Drug category	Stress fracture, *n*			Ankle sprain (control), *n*			*p* value	Significance
	Yes	No	Not sure	Yes	No	Not sure		
ASPIRIN3M	9	0	0	4	0	0	>0.99	ns
ADVIL3M	20	4	0	5	6	0	0.04	^∗^
ACETOCT3M	9	1	0	1	2	0	0.11	ns
ACETPR3M	4	0	0	1	0	0	>0.99	ns
All drug categories 3M	42	14	0	11	39	0	<0.0001	^∗∗∗∗^

This table includes the enumerated responses of subjects within each injury group regarding regular drug use defined as three or more times per week for the three months prior to the time of inquiry. ^∗^*p* < 0.05; ns = not significant. ^∗∗∗∗^ is defined as significant with a *p* value <0.0001.

## Data Availability

All data generated or analyzed in this study are included in this published article and its supplemental data file.

## Supplementary Materials

Supplementary Materials Supplementary materials are provided in the attached file titled “Advances in Orthopedics_NSAID Stress Fracture_Supplemental Materials.docx.” This file includes Supplementary Figure 1 that has a CONSORT flow diagram detailing the subject selection process.
