# Supplementary figures and images for: Regular Nonsteroidal Anti-Inflammatory Drug Use Increases Stress Fracture Risk in the General Population: A Retrospective Case-Control Study
Source: Adv Orthop. 2024 Oct 12;2024:7933520. doi: 10.1155/2024/7933520 (PMC11490349; doi:10.1155/2024/7933520)

**SUPPLEMENTAL DATA**


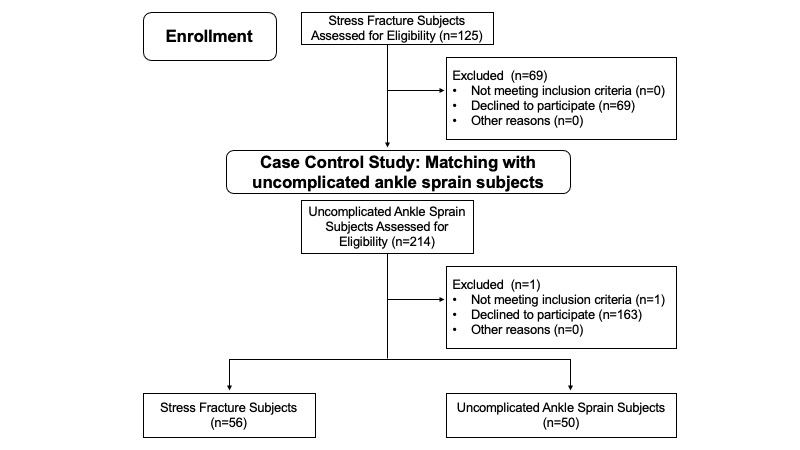


**Supplementary Figure 1.** CONSORT Flow Diagram. Subject selection process.

Supplement: Supplementary Materials — are provided in the attached file titled “Advances in Orthopedics_NSAID Stress Fracture_Supplemental Materials.docx.” This file includes Supplementary Figure 1 that has a CONSORT flow diagram detailing the subject selection process. [file 7933520.f1.docx]
